# Factors related to retinal nerve fiber layer thickness in bipolar disorder patients and major depression patients

**DOI:** 10.1186/s12888-021-03270-7

**Published:** 2021-06-10

**Authors:** Yanhong Liu, Yongsheng Tong, Lvzhen Huang, Jingxu Chen, Shaoxiao Yan, Fude Yang

**Affiliations:** 1 Department of Psychiatry, Peking University Huilongguan Clinical Medical School, Nandian Road, Changping District, Beijing, 100096 China; 2 Department of Psychiatry, Beijing Huilongguan Hospital, Beijing, China; 3 Department of Ophthalmology, People’s Hospital of Peking University, Beijing, China

**Keywords:** Bipolar disorder, Major depression, Retinal nerve fiber layer, Thickness, Metabolism

## Abstract

**Background:**

We analyzed the correlation of the clinical data with retinal nerve fiber layer (RNFL) thickness and macular thickness in bipolar disorder patients and major depression patients. The aim of this study is to explore factors that affect RNFL thickness in bipolar disorder patients and major depression patients, with a view to providing a new diagnostic strategy.

**Methods:**

Eighty-two bipolar disorder patients, 35 major depression patients and 274 people who were age and gender matched with the patients were enrolled. Demographic information and metabolic profile of all participants were collected. Best-corrected visual acuity of each eye, intraocular pressure (IOP), fundus examination was performed. RNFL and macular thickness were measured by optical coherence tomography (OCT). Correlations between RNFL and macular thickness and other data were analyzed.

**Results:**

RNFL and macula lutea in bipolar dipolar patients and major depression patients are thinner than normal people. Triglyceride and UA levels are the highest in the bipolar disorder group, while alanine aminotransferase (ALT) and glutamic oxalacetic transaminase (AST) levels in the depression group are the highest. Age onset and ALT are positively while uric acid (UA) is negatively correlated with RNFL thickness in bipolar dipolar patients. Cholesterol level is positively correlated with RNFL thickness while the duration of illness is correlated with RNFL thickness of left eye in major depression patients.

**Conclusions:**

RNFL and macula lutea in bipolar dipolar patients and major depression patients are thinner than normal people. In bipolar disorder patients, age-onset and ALT are potential protective factors in the progress of RNFL thinning, while UA is the pathological factor.

**Supplementary Information:**

The online version contains supplementary material available at 10.1186/s12888-021-03270-7.

## Background

Bipolar disorder and major depression are two psychiatric diseases that bring great burden to both patients and society [1, 2]. Bipolar disorder is also known as manic-depressive disorder; its manifestations include extreme shifts in mood (depression and mania), energy and activity, which are more severe than the ups and downs in the mood of normal people and can thus affect patients’ ability of completing normal daily tasks [3]. Its global prevalence is estimated to be 2.4%, such a high rate that the disease should be taken seriously by people [4]. Major depression is defined by having these symptoms every day for at least 2 weeks: a depressed mood during most times of the day, and loss of interest in normal activities and relationships [5]. The global prevalence of major depression is approximately 11% [6]. These two diseases have greatly affected patients’ life [1, 2].

However, despite the development of drugs to treat these two diseases [7, 8], there are still many questions about them. One problem is that we are still not fully aware of their pathogenic factors, despite the fact that genetic and environmental factors have been proved to be related to disease onset [9, 10]. And due to the lack of evidence of specific pathogenic factors, it’s difficult to detect the early onset of bipolar disorder and major depression, and most patients are diagnosed when the diseases already break out [1, 2]. Another question is the accompanying symptoms and the prognosis of bipolar disorder and major depression. It’s been reported that patients’ retina may display abnormal changes. For example, the thickness of the entire retina in the macula lutea area, as well as the thickness of the retinal nerve fiber layer (RNFL) on the edge of the retina are shown to be thinner in bipolar disorder patients [11, 12]. Also, a new study shows that retinal vascular trajectory is a potential marker for BD [13, 14], and the duration of the depressive episode is correlated with RNFL thickness [15]. Nevertheless, whether such optical changes can be used in the diagnosis of the two diseases, as well as the factors that affect these optical changes, are still unknown. However, since the axons in RNFL are nonmyelinated, its pathological changes have been used in the visualization of some neurodegenerative diseases, for example, Alzheimer’s Disease and Parkinson’s Disease, and it’s been found that the thinner RNFL is possibly related to the decrease cognitive abilities in these patients [16], which make it possible to also use these optical changes as potential markers of bipolar disorder and major depression.

Therefore, considering the above unresolved questions, we conducted this study to explore the factors that could affect the optical changes in bipolar disorder and major depression patients, so as to provide evidence for a novel diagnostic strategy of these two diseases.

## Methods

### Participants

In this cross-sectional study, 82 bipolar disorder patients and 35 major depression patients were enrolled, including the patients treated in psychiatric outpatient clinics and the patients treated in hospital, and they were categorized as Bipolar disorder group and Depression group, respectively. In addition, 274 people who were age and gender matched with the patients were enrolled as Normal Control group.

Inclusion criteria were as follows: (1) For patients, bipolar disorder and major depression were diagnosed by The Structured Clinical Interview for Diagnostic and Statistical Manual of Mental Disorders Fourth Version (DSM-IV) (SCID) [1, 2]. The patients with bipolar disease were enrolled in the stable state either depression phase or manic phase or mixed. The patients all were in stable state. (2) For patients, the doses of antipsychotics should remain unchanged during the trial. (3) For patients, benzodiazepines and anticholinergic should be avoided during the trial. (4) Normal people were deemed as normal by DSM-IV. (5) The patients or their legal guardians agreed to participate in this trial and signed written informed consent. Exclusion criteria were as follows: (1) Had ocular diseases, including macular degeneration, optic neuropathy, glaucoma, eye traumas, or other disease that could affect the structure of RNFL. (2) Had eye surgery during the past 3 months before enrollment. (3) Had a history of hypotension crisis, or other intracranial diseases or intraorbital space-occupying lesion that could affect the visual pathway. (4) Had diabetes mellitus. (5) Had cerebrovascular diseases, including cerebral hemorrhage and cerebral infarction. (6) Had grade 2 or greater hypertension. (7) Had thyroid dysfunction or other metabolic diseases.

### Methods

#### General information and clinical exams

Smoking information during the past 3 months before enrollment was collected. Weight, height and blood pressure were measured. The blood sample was collected at 6 A.M. before breakfast, and biochemical parameters were analyzed, including uric acid, blood lipids, thyroid function tests, etc.

Patients’ cognitive functions were measured by the Repeatable Battery Neuropsychological Status (RBANS).

#### Ophthalmologic examinations

Best corrected visual acuity of each eye was evaluated by Snellen visual chart. Intraocular pressure (IOP) was measured by slit lamp microscopy of the Goldman tonometer. Fundus examination was performed with 90D ophthalmic condensing lens, including the thickness of peripapillary RNFL and the macula lutea.

Optical coherence tomography was conducted using OCT 20002 (Topcorn, Japan). The intensity of the scanning signal ≥7 was considered acceptable. Rester scan of the peripapillary RNFL was conducted in the 6 mm × 6 mm peripapillary area by using the “200×200 optic disk volumetric scan” module. The center of the was determined by the software, and then the thickness of peripapillary RNFL was measured in the peripapillary circle (diameter = 3.4 mm). The thickness of RNFL in the four quadrants (superior, inferior, nasal and temporal) and 12 clock positions of the entire eyeball were measured, and mean thickness (mm) was calculated. Rester scan of the macula lutea was conducted in the 6 mm × 6 mm peripapillary area by using the “512×128 macular volumetric scan” module, which can qualitatively and quantitatively evaluate the thickness of retina (mm).

### Statistical analysis

Data were analyzed by SPSS 20.0. Normal distribution was tested by Shapiro Wilk test. Data with normal distribution were analyzed by independent t-test and/or one-way ANOVA. The thickness of RNFL, thickness and volume of macula lutea among different groups were analyzed by multivariate analysis of covariance. Data were also analyzed among sub-groups by Least-Significant Difference method. *P* < 0.05 was considered significantly different.

## Results

### Demographic data of the participants

As shown in Table 1, for the 82 patients with bipolar disorder, 35 patients with major depression, and 274 normal people, there’s no significant difference in age (*P* = 0.079) or gender proportion (*P* = 0.634) among the three groups. The duration of illness is not significantly different between the bipolar disorder group and the depression group (*P* = 0.107). The positive rate of family history of the mental disease was significantly different among the three groups (*P* < 0.001), with the bipolar disorder group being the highest (29.3%), and the depression group was the second highest (5.71%), and the normal people had the lowest positive rate (1.1%). The numbers of smokers were also significantly different among the three groups (*P* = 0.020), and the proportion of smoking participants in the bipolar disorder group was the highest (14.6%).

**Table 1:** Demographic, metabolic and basic optical data of the participants

Mean ± SD	Bipolar disorder group	Depression group	Normal Control group	t/F/χ^2^	*P*
Age (year)	38.96 ± 14.61	43.60 ± 15.51	41.59 ± 9.37	2.558	0.079
Gender Female (n)	64	30	220	0.912	0.634
Male (n)	18	5	54		
Duration of illness (year)	13.27 ± 11.44	8.43 ± 9.55	–	1.636	0.107
Smokers, n (%)	12 (14.6)	2 (5.7)	12 (4.4)	16.737	0.020
Positive family history, n (%)	24 (29.3)	2 (5.71)	3 (1.1)	32.715	< 0.001
BMI	24.68 ± 3.65	24.32 ± 4.86	23.94 ± 3.34	1.165	0.313
SBP^a^ (mmHg)	109.88 ± 8.39	108.86 ± 9.32	109.23 ± 9.22	0.213	0.808
DBP^b^ (mmHg)	71.28 ± 5.76	69.43 ± 5.39	70.69 ± 5.84	1.257	0.286
MAP^c^ (mmHg)	84.14 ± 6.19	82.57 ± 6.27	83.54 ± 6.38	0.778	0.460
ALT^d^ (U/L)	16.66 ± 13.33	33.26 ± 30.91	20.16 ± 15.14	10.423	< 0.001
AST^e^ (U/L)	18.52 ± 10.35	24.97 ± 11.44	19.31 ± 7.89	6.403	0.002
Glucose (mmol/L)	5.34 ± 2.70	4.67 ± 0.37	5.43 ± 1.51	2.240	0.108
Cholesterol (mmol/L)	4.24 ± 0.98	4.08 ± 0.74	4.77 ± 0.84	15.644	< 0.001
Triglyceride (mmol/L)	1.96 ± 1.22	1.64 ± 0.85	1.50 ± 1.24	3.857	0.022
UA^f^ (mol/L)	359.90 ± 72.50	279.90 ± 75.54	321.63 ± 83.80	11.548	< 0.001
Thyrotropin (mIU/L)	2.97 ± 2.43	2.29 ± 1.28	1.95 ± 0.81	1.585	0.212
TT3^g^ (nmol/L)	0.90 ± 0.21	0.81 ± 0.21	0.96 ± 0.18	2.078	0.133
FT3^h^ (pmol/L)	3.23 ± 0.66	3.19 ± 0.93	4.17 ± 1.49	5.685	0.005
TT4^i^ (nmol/L)	12.76 ± 22.32	10.15 ± 15.61	7.64 ± 2.61	0.386	0.681
FT4^j^ (pmol/L)	1.50 ± 0.15	1.17 ± 1.97	0.83 ± 0.13	0.399	0.673
Eye pressure left (mmHg)	14.74 ± 3.22	13.97 ± 3.09	15.39 ± 2.70	4.303	0.014
Right (mmHg)	14.67 ± 3.53	14.30 ± 2.35	15.24 ± 2.51	2.419	0.091
Diopters (D) left eye (unnormal,n)	4.10 ± 1.74 (29)	2.60 ± 0.55 (5)	2.68 ± 1.18 (15)	5.322/12.00	0.008 (0.285)
right eye (unnormal,n)	4.12 ± 1.77 (29)	2.1 ± 0.52 (5)	2.71 ± 1.32 (15)	6.086/12.00	0.005 (0.285)
RBANS					
Immediate memory	75.10 ± 20.49	72.52 ± 13.87	106.84 ± 10.51	151.15	< 0.001
Visuospatial/Constructional	95.10 ± 14.81	95.92 ± 14.71	105.69 ± 14.91	14.08	< 0.001
Language Index	90.91 ± 18.92	84.44 ± 14.78	106.82 ± 9.55	53.63	< 0.001
Attention Index	97.07 ± 12.41	101.92 ± 11.08	120.04 ± 16.56	60.71	< 0.001
Delayed Memory	86.10 ± 13.58	85.56 ± 16.65	111.14 ± 7.86	158.32	< 0.001
Total Scale(t)	84.84 ± 13.78	83.24 ± 12.64	117.64 ± 12.13	198.91	< 0.001

^a^Systolicblood pressure

^b^Diastolyc blood pressure

^c^Mean arterial pressure

^d^Alanine aminotransferase

^e^Aspartate aminotransferase

^f^Uric acid

^g^Serum total thyroid hormone 3

^h^Free thyroid hormone 3

^i^Serum total thyroid hormone 4

^j^Free thyroid hormone 4

### Metabolic and basic optical data of the participants

Also shown in Table 1, BMI (*P* = 0.313), SBP (*P* = 0.808), DBP (*P* = 0.286), MAP (*P* = 0.460), glucose (*P* = 0.108), four thyroid function indexes (thyrotropin: *P* = 0.212; TT_3_: *P* = 0.133; TT_4_: *P* = 581; FT4: *P* = 673) are not significantly different among the three groups, while the level of FT_3_ shows significant difference (*P* = 0.005) and is lower in bipolar disorder patients and depression patients. However, the levels of ALT (*P* < 0.001), AST (*P* = 0.002), UA (*P* < 0.001), triglyceride (*P* = 0.022) and cholesterol (*P* < 0.001) are significantly different among the three groups, and the levels of triglyceride (1.96 ± 1.22 mmol/L) and UA (359.90 ± 72.50 mol/L) show the highest in the bipolar disorder group, while the levels of ALT (33.26 ± 30.91 U/L) and AST (24.97 ± 11.44 U/L) in the depression group are the highest.

Among the three groups, there’s no significant difference in right eye pressure (*P* = 0.091), but left eye pressure is significantly different (*P* = 0.014) and is lower in patients than in normal people. In addition, diopters of each eye are also significantly different among the three groups (right eye: *P* = 0.005; left eye: *P* = 0.008), with bipolar patients having the highest diopters.

### RNFL thickness and macular thickness in different groups

**Fig. 1:**
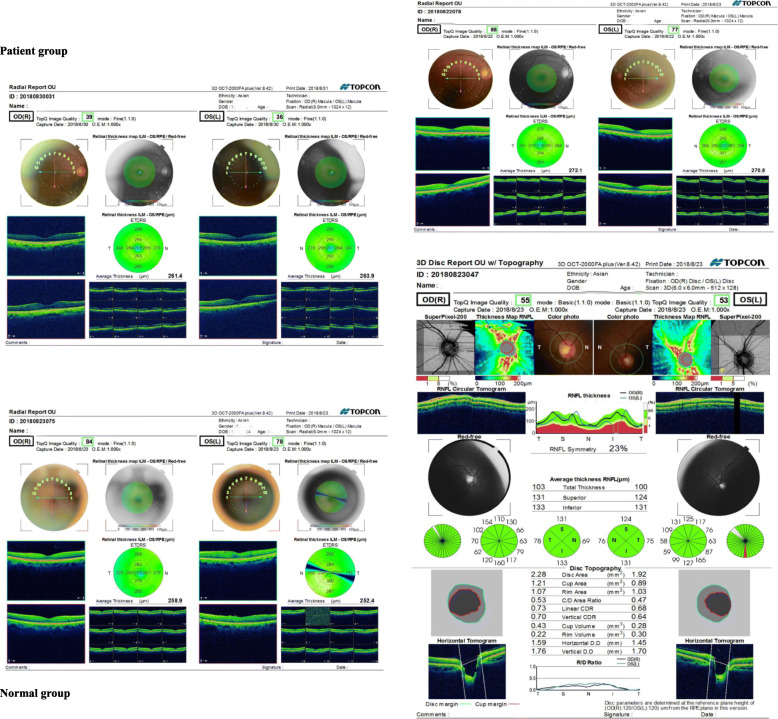
The representative optical coherence tomographic OCT structure images

Figure 1 shows the representative optical coherence tomographic OCT structure images. As shown in Table 2, total thickness (left eye: *p* = 0.001; right eye: *p* = 0.004) and superior thickness (left eye: *p* = 0.010; right eye: *p* = 0.017) of both eyes were significantly different among the three groups, with results in the bipolar disorder group being the thinnest. For inferior thickness, among the three groups, there’s no significant difference in the right eye (*P* = 0.823) but is significantly different in the left eye (*P* = 0.010). For disc area (right eye: *p* = 0.202; left eye: *p* = 0.224) and cup volume (right eye: *p* = 0.934; left eye: *p* = 0.252), there’s also no significant difference among the three groups in both eyes.

**Table 2:** RNFL thickness and macular thickness in different groups

Thickness (Mean ± SD)	Bipolar disorder group	Depression group	Normal Control group	F	P/p (post-hoc)	P′
Right RNFL						
Total thickness (μm)	90.69 ± 19.97	100.18 ± 14.12	102.13 ± 14.32	12.29	< 0.001/< 0.001^a^/0.593^b^/0.016^c^	0.004
Superior thickness (μm)	110.34 ± 29.22	123.59 ± 23.53	120.13 ± 14.32	3.14	0.045/0.022^a^/0.607^b^/0.067^c^	0.017
Inferior thickness (μm)	116.72 ± 33.32	122.05 ± 23.05	130.17 ± 22.37	6.75	0.001/< 0.001^a^/0.168^b^/0.401^c^	0.823
Disc area (mm^2^)	2.35 ± 1.15	2.62 ± 0.63	2.34 ± 0.67	1.17	0.313	0.202
Cup volume (mm^3^)	0.10 ± 0.12	0.26 ± 0.24	0.11 ± 0.13	11.55	< 0.001/0.697^a^/< 0.001^bc^	0.934
Left RNFL						
Total thickness (μm)	91.61 ± 14.85	97.27 ± 16.71	103.71 ± 11.94	19.73	< 0.001/< 0.001^a^/0.033^b^/0.084^c^	0.001
Superior thickness (μm)	115.13 ± 25.36	129.95 ± 21.32	128.55 ± 22.51	8.12	< 0.001/< 0.001^a^/0.788^b^/0.01^c^	0.010
Inferior thickness (μm)	119.13 ± 26.78	115.45 ± 34.02	133.88 ± 18.68	13.65	< 0.001/< 0.001^ab^/0.513^c^	0.010
Disc area (mm^2^)	2.24 ± 1.17	2.32 ± 0.64	2.24 ± 0.57	0.11	0.899	0.224
Cup volume (mm^3^)	0.08 ± 0.11	0.18 ± 0.12	0.11 ± 0.13	5.16	0.006/0.150^a^/0.011^b^/0.001^c^	0.252
Right macular (μm)						
Average thickness	255.24 ± 14.47	287.34 ± 26.01	263.82 ± 41.93	1.79	0.168	0.688
Central thickness	220.06 ± 48.22	215.67 ± 25.45	227.03 ± 23.87	3.10	0.046	0.218
Inner superior	273.49 ± 70.54	271.97 ± 71.62	302.54 ± 16.22	20.45	< 0.001/< 0.001^ab^/0.855^c^	0.001
Inner inferior	263.52 ± 68.68	257.86 ± 70.50	293.47 ± 19.67	23.55	< 0.001/< 0.001^ab^/0.499	0.001
Inner nasal	271.97 ± 71.02	271.03 ± 70.95	298.85 ± 30.97	13.33	< 0.001/< 0.001^a^/0.001^b^/0.921	0.001
Inner temporal	262.11 ± 76.56	255.37 ± 66.42	287.27 ± 15.86	16.94	< 0.001/< 0.001^ab^/0.431	< 0.001
Outer ring superior	246.61 ± 62.93	241.34 ± 63.01	267.60 ± 14.81	15.72	< 0.001/< 0.001^ab^/0.739	0.013
Outer inferior	234.05 ± 61.13	236.46 ± 61.03	255.79 ± 15.11	13.73	< 0.001/< 0.001^a^/0.002^b^/0.739^c^	0.005
Outer nasal	257.19 ± 65.72	257.49 ± 66.69	283.07 ± 16.44	17.69	< 0.001 < 0.001^ab^/0.970	0.002
Outer temporal	225.22 ± 61.27	226.51 ± 58.73	248.99 ± 16.62	16.83	< 0.001/< 0.001^ab^0.858^c^	0.002
Left macular (μm)						
Average thickness	253.15 ± 48.47	266.40 ± 18.28	263.91 ± 36.37	2.53	0.081/0.033^a^/0.722^b^/0.097^c^	0.858
Central thickness	221.58 ± 45.12	225.85 ± 28.21	224.19 ± 21.71	0.33	0.718	0.620
Inner superior	285.56 ± 54.35	295.82 ± 20.27	302.96 ± 17.16	10.69	< 0.001/< 0.001^a^/0.177^b^/0.089^c^	0.038
Inner inferior	274.90 ± 51.80	287.97 ± 28.25	292.06 ± 20.18	9.45	< 0.001/< 0.001^a^/0.456^b^/0.037^c^	0.515
Inner nasal	286.60 ± 54.18	293.18 ± 27.48	298.44 ± 26.10	3.63	0.027/0.008^a^/0.396^b^/0.351^c^	0.700
Inner temporal	270.56 ± 53.12	271.39 ± 25.46	282.70 ± 18.62	6.24	0.002/0.002^a^/0.037^b^/0.891^c^	0.081
Outerring superior	248.63 ± 52.68	268.39 ± 16.24	267.11 ± 17.73	12.83	< 0.001/< 0.001^a^/0.804^b^/0.001^c^	0.096
Outer inferior	241.28 ± 50.35	252.82 ± 21.51	255.43 ± 18.16	7.39	0.001/< 0.001^a^/0.610^b^/0.048^c^	0.094
Outer nasal	264.64 ± 50.12	277.97 ± 23.37	279.0 ± 25.18	5.96	0.003/0.001^a^/0.859^b^/0.045^c^	0.174
Outer temporal	235.64 ± 48.95	248.03 ± 18.28	247.96 ± 18.87	5.89	0.003/0.001^a^/0.989^b^/0.032^c^	0.605

P′: adjusted p for analysis of covariance after controlling for smoker, ALT, AST, Glucose, Cholesterol, Triglyceride, Uric acid (left eye also controlling for left eye pressure) with Ancova

P (post-hoc)

^a^comparison between Bipolar disorder group and Normal Control group

^b^comparison between Depression group and Normal Control group

^c^comparison between Bipolar disorder group and Depression group

When compared among the three groups, there’s no significant difference in average macular thickness (right eye: *p* = 0.688; left eye: *p* = 0.858) or central macular thickness (right eye: *p* = 0.218; left eye: *p* = 0.620) in both eyes. The inner (superior: *P* = 0.001; inferior: *P* = 0.001; nasal: *P* = 0.001; temporal: *P* < 0.001) and outrings (superior: *P* = 0.013; inferior: *P* = 0.005; nasal: *P* = 0.002; temporal: *P* = 0.002) thickness of right macular in all four quadrants are significantly different among the three groups. For left eye, innersuperior is significantly different among the three groups (*P* = 0.038), and innersuperior and outsuperior of the bipolar disorder group macular thickness are the thinnest. In addition, all parameters of macular thickness in the bipolar disorder group are thinner than the normal control group (See Supplement files 1 and 2).

### Analysis of the factors related to RNFL and macular thickness in bipolar disorder and major depression patients

As shown in Table 3, after controlling the factor of smoking, age, sex, BMI, MAP, diopter, glucose, cholesterol and triglyceride, age-onset, UA and ALT are shown to be the factors that are significantly related to the RNFL and macular thickness. When age-onset increases, the degree of RNFL atrophy becomes less. Similarly, with the increase of ALT level, macular also becomes thicker. On the contrary, when UA level increases, macular thickness decreases. Another finding is that the above relationship between age onset, UA, ALT and RNFL or macular thickness are almost limited to the right eye, as only the inner inferior thickness of the left macular is significantly related to ALT level.

**Table 3:** The factors related to RNFL and macular thickness in bipolar disorder patients^a,g^

Right RNFL	Constant	Age of onset				UA				ALT			
		ßl	S.E.	F1	P1	ß2	S.E.	F2	P2	ß3	S.E.	F3	P3
Total thickness (μm)	115.56	0.614	0.241	2.549	0.018								
Superior thickness (μm)^b^	64.02	1.070	0.384	2.787	0.010								
Inferior thickness (μm)^c^	284.81	0.820	0.403	2.037	0.054	0.180	0.072	2.488	0.021	−0.952	0.283	−3.358	0.003
Disc area (mm^2^)^d^	−6.057	0.050	0.021	2.373	0.028								
Right macular (μm)													
Inner superior^e^	269.46	−2.337	0.994	−2.351	0.027	−0.350	0.161	− 2.178	0.039	2.244	0.741	3.030	0.006
Inner inferior	307.51	−2.240	1.051	−2.131	0.044	−0.383	0.170	−2.255	0.034	1.195	0.783	2.548	0.018
Inner nasal^e^	186.60	−1.943	0.983	−1.976	0.060	−0.043	0.155	−2.604	0.016				
Inner temporal	290.80	−2.214	0.914	−2.323	0.029	−0.411	0.148	−2.782	0.010	1.926	0.681	2.829	0.009
Outer ring superior^e^	145.50	−1.938	0.829	−2.339	0.028	−0.339	0.130	−2.605	0.016	1.528	0.647	2.363	0.027
Outer inferior^e^	191.52	−1.711	0.855	−2.001	0.057	−0.277	0.138	−2.008	0.057	1.884	0.629	2.994	0.006
Outer nasal^e^	126.94	−1.583	0.862	−1.838	0.080	−0.333	0.136	−2.444	0.023	1.408	0.673	2.093	0.048
Outer temporal^e^	163.11	−1.737	0.869	−1.999	0.058	−0.364	0.137	−2.668	0.014	1.324	0.678	1.952	0.063
Left RNFL													
Total thickness (μm)	116.03					−0.061	0.036	−1.705	0.099				
Left macular													
Inner superior	292.67									0.208	0.120	1.735	0.093
Inner inferior^f^	313.12									0.239	0.108	2.219	0.034

ß: Beta of the multiple models, showing the variation of dependent variables (Y-axis) following an increase in the independent variable (X-axis)

*ALT* alanine aminotransferase, *AST* glutamic oxalacetic transaminase, *UA* uric acid, *S.E* Standard error

^a^Linear regression with using smoker, age onset, sex, duration of illness (DOI), Family history, MAP, BMI, ALT, Glucose, Cholesterol, UA, FT3, eye pressure as independent variables

^b^FT3 as covariates significantly in the equation

^c^Family history and MAP as covariates significantly in the equation

^d^Glucose, BMI, Sex as covariates significantly in the equation

^e^Smoker as a covariate significantly in the equation

^f^Smoker and sex as covariates significantly in the equation

^g^all the unlisted variables of retina above have no significant affective factors to enter the equation

The data of major depression are shown in Table 4. It can be seen that after controlling the factor of smoking, age, sex, BMI, MAP, diopter, glucose, UA and triglyceride, duration of illness, the level of cholesterol and ALT are the factors that are significantly related to the RNFL and macular thickness in major depression patients. Although age-onset showed nearly no significant relationship with RNFL thickness, when the duration of illness prolongs, RNFL becomes thinner, but this is mainly found in the left eye. When the level of cholesterol increases, the inferior thickness of RNFL in both eyes and the total thickness of RNFL in the left eye are thicker. The relationship between ALT and macular thickness of both eyes is different. When the ALT level increases, the inner and outer macular thickness of the right eye decreases, but the nasal and temporal inner-macular thickness of the left eye also increases with the ALT level. In conclusion, the significant relationships between RNFL and macular thickness and three factors in major depression are limited to either right or left eye.

**Table 4:** The factors related to RNFL and macular thickness in major depression patients^a^

Right RNFL	Constant	Age onset^b^ /duration of illness^c^				CHO				ALT			
		ßl	S.E.	F1	P1	ß2	S.E.	F2	P2	ß3	S.E.	F3	P3
Total thickness (μm)	86.34	−0.602	0.282	−2.185	0.059^b^								
Inferior thickness (μm)	−25.28	−1.268	0.574	−2.211	0.058^b^	26.62	9.232	2.884	0.020				
Right macular (μm)													
Average thickness ^d^	352.80	0.630	0.293	2.155	0.050^b^	−13.204	5.203	−2.538	0.020				
Inner superior	312.09									−1.094	0.499	− 2.191	0.042
Ourter superior	277.77									−1.042	0.432	−2.410	0.027
Outer inferior	270.70									−0.995	0.422	−2.357	0.030
Left RNFL													
Total thickness (μm)^e^	−68.81	− 0.145	0.019	−7.532	0.002^c^	17.438	2.835	6.152	0.004				
Inferior thickness (μm)^f^	−170.57	−0.249	0.044	−5.655	0.002^c^	28.310	6.264	4.519	0.006				
Disc area (mm^2^)^g^	8.805	−0.033	0.008	−4.322	0.023								
Cup volume (mm^3^)^h^	−0.874	−0.002		−8.904	< 0.001^c^					0.008	0.001	8.261	< 0.001
Left macular (μm)													
Inner superior	284.25	0.366	0.143	2.560	0.020^b^								
Inner inferior													
Inner nasal	279.40									0.440	0.193	2.278	0.036
Inner temporal	261.01									0.396	0.168	2.354	0.031
Ourter superior	222.29	−0.104	0.048	−2.166	0.047^c^								

^a^ linear regression with using smoker, age onset, sex, duration of illness, BMI, MAP, ALT, Glucose, Cholesterol, UA, FT3, as independent variables

*ALT* alanine aminotransferase, *AST* glutamic oxalacetic transaminase, *UA* uric acid, *CHO* Cholesterol, *S.E* Standard error

^b^Age onset

^c^Duration of illness

^d^UA as covariates significantly in the equation

^e^Smoker, Sex, BMI,MAP, FT3,Glucose as covariates significantly in the equation

^f^Sex, BMI,BMP, FT3,Glucose as covariates significantly in the equation

^g^Sex, FT3,UA, Glucose as covariates significantly in the equation

^h^Smoker, Sex,MAP, Glucose as covariates significantly in the eq

^i^BMI as covariates significantly in the equation

## Discussion

The hypotheses of our study are such that the change in RNFL thickness of bipolar patients is directly related to their disease onset age, while in depression patients, the change in RNFL thickness is just one of the symptoms of depression patients. In order to prove that, we compared the clinical data and the thickness and macular thickness of bipolar disorder patients, major depression patients and normal people. Then we analyzed the correlation of the clinical data with RNFL thickness and macular thickness in bipolar disorder patients and major depression patients. There’s no significant difference in demographic data among the three groups, except family history of the mental disease that’s higher in bipolar disorder patients, which is the characteristics of this disease [1]. This result means that the enrolled patients were comparable as their basic data were similar.

For the metabolic data in all participants, FT_3_ is significantly lower in bipolar disorder patients and major depression patients, which is also in consistent with previous literature [1, 2]. In addition, the levels of triglyceride and UA are the highest in the bipolar disorder group, while the levels of ALT and AST in the depression group are the highest. These results indicate that there are indeed differences in metabolic profiles between patients and normal people. In previous studies, it’s been reported that blood lipid and their metabolites of bipolar patients are different from normal people, which could be due to the disorder in mitochondria functions [7, 17]. And it’s also reported that most cases of major depression could be due to the dysregulated metabolic systems in patients [18]. However, it’s not yet reported that the hepatic function of bipolar disorder patients and major depression patients would worsen, thus causing the rise of ALT and AST levels. We speculated that the higher ALT and AST levels in depression patients could be due to the effects of anti-depressants, however, it needs further exploration to confirm.

We continued to explore the differences in optical parameters and found that left eye pressure is significantly lower in patients than in normal people, while there’s no significant difference in right eye pressure. Nevertheless, all eye pressure data are within normal range, so it’s no considered a clinically significant parameter and was not taken into analysis by use. In addition, diopters of each eye are significantly higher in bipolar disorder patients and has reached the range of hyperopia. However, there’s no such reports yet and thus we speculated that this phenomenon could be due to the pathological changes in patients’ eyes, but further studies are needed to fully elucidate the reasons.

We then explored which parameters are related to these two diseases. We found that in bipolar disorder patients, with the increase of the age-onset, the degree of RNFL atrophy showed less than that of early onset. Therefore, the late onset of bipolar disorder is a potential protective factor of the RNFL thickness. Similarly, ALT was also shown to have potential protective effects on macular thickness of bipolar disorder patients. However, following the increase of the level of UA, the macular became thinner, suggesting that UA could be a factor contributing to the worsening of RNFL thinning. Thus, we concluded that in bipolar disorder patients, age-onset and ALT are potential protective factors in the progress of RNFL thinning, while UA is the pathological factor. And the data also show that RNFL thickness of bipolar patients are directly related to disease onset age, indicating that RNFL thickness was changed when the disease initially began, and thus the change in RNFL thickness is more likely due to the genetic factors of the patients, not other factors appear during the development of the disease, which makes it possible to be used as a biomarker for bipolar disorder.

In major depression patients, we found that duration of illness could contribute to the thinning of RNFL, while cholesterol level could be protective. Interestingly, when the ALT level increases, the inner and outer macular thickness of the right eye decreases, but the nasal and temporal inner-macular thickness of the left eye also increases with the ALT level. Thus, ALT is ruled out due to its difference in each eye, and we concluded that cholesterol level and duration of illness could be used in the prognosis of RNFL thinning in major depression patients. It’s also reported that RNFL is thinner in metabolic syndrome patients, and it could be due to the degeneration of nerve fibers in these patients [19]. In addition, the retinal nerve fiber layer is composed of retinal ganglion cell exons, which is homologous with the central neural system in the process of embryonic development [20], and researchers have found that the thickness of RNFL is negatively correlated with the cognitive abilities of patients with degenerative neural diseases [21, 22]. Thus, we can speculate that the change in UA and ALT levels in bipolar disorder patients, and the change in cholesterol and ALT levels in depression patients, can be used to predict the progress and prognosis of the disease. Also, for patients with different levels of UA, ALT and cholesterol, different treatments may be used considering the side effects of drugs and the disease progress of patients. As some metabolic parameters changed in depression patients, which although have not reached the extent of metabolic diseases and are still within normal range but could cause the change in RNFL thickness. And considering that the metabolic changes are commonly seen in depression patients, it can be concluded that for depression patients, the change in RNFL thickness is just one of the symptoms during the disease of depression.

We also found that RNFL of depression patients could affect patients’ attention. Although it is a new finding that needs further evidence to support, we suggest that the possible explanation could be that the thinned RNFL resulted in optical changes, which subsequently affect patients’ attention.

However, the clinical medication of patients and normal controls were observed in a natural state, and there may be the effect of bias in medication between groups. It should be noted that the use of antidepressant and mood stabilizers is not controlled, that is, patients continued using these drugs during the trial. Therefore, the drugs could exert certain effects on the results of the trial. In addition, this is a cross-sectional study, so the conclusions were based on the results of multivariate analysis of covariance and linear regression equation, so it needs confirmation in future clinical trials. For the patients who were all in the stable stage of the disease, the relationship between the severity and the results was not to be studied.

## Conclusions

We found that RNFL and macula lutea in bipolar disorder patients and major depression patients are thinner than normal people. In bipolar disorder patients, age-onset and ALT are potential protective factors in the progress of RNFL thinning, while UA is the pathological factor. In major depression patients, cholesterol level is positively correlated with RNFL thickness, and cholesterol level and duration of illness could be used in the prognosis of RNFL thinning. RNFL thickness of bipolar patients is related to disease onset age, while in depression patients, the change in RNFL thickness is just one of the symptoms.

## Supplementary Information

**Additional file 1.** RNFL and macula lutea) Group specificity, sensitivity, accuracy and ROC analysis.

**Additional file 2.** Major depression patient groups specificity, sensitivity, accuracy and ROC analysis.

## Data Availability

All data generated or analyzed during this study are included in this published article.

## Abbreviations

RNFL: Retinal nerve fiber layer
IOP: Intraocular pressure
DSM-IV: Disorders Fourth Version
RBANS: Repeatable Battery Neuropsychological Status
