# Supplementary material for: Factors related to retinal nerve fiber layer thickness in bipolar disorder patients and major depression patients
Source: BMC Psychiatry. 2021 Jun 10;21:301. doi: 10.1186/s12888-021-03270-7 (PMC8191183; doi:10.1186/s12888-021-03270-7)
Supplement: Supplementary file 1 — Additional file 1. RNFL and macula lutea) Group specificity, sensitivity, accuracy and ROC analysis. [file 12888_2021_3270_MOESM1_ESM.docx]

Biphasic:


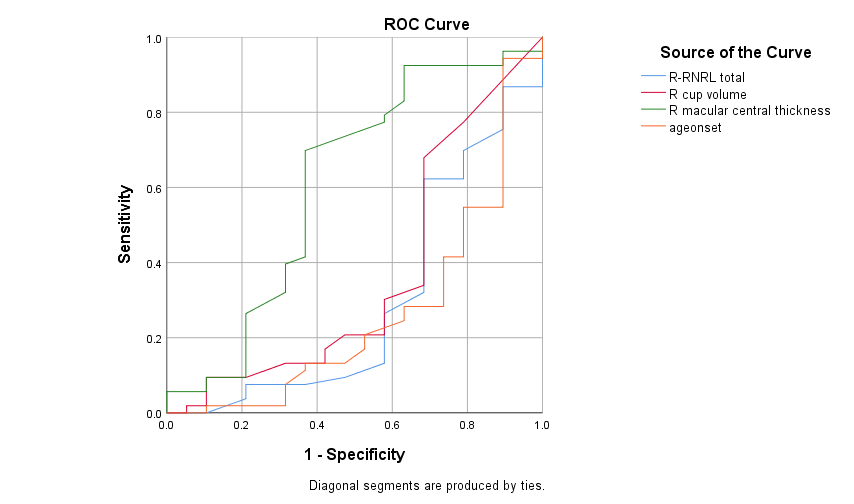


| **Area Under the Curve** | |
| --- | --- |
| Test Result Variable(s) | Area |
| R-RNRL total | .299 |
| R cup volume | .365 |
| R macular central thickness | .610 |
| ageonset | .263 |
| The test result variable(s): R-RNRL total, R cup volume, R macular central thickness, ageonset has at least one tie between the positive actual state group and the negative actual state group. Statistics may be biased. | |

Biphasic:


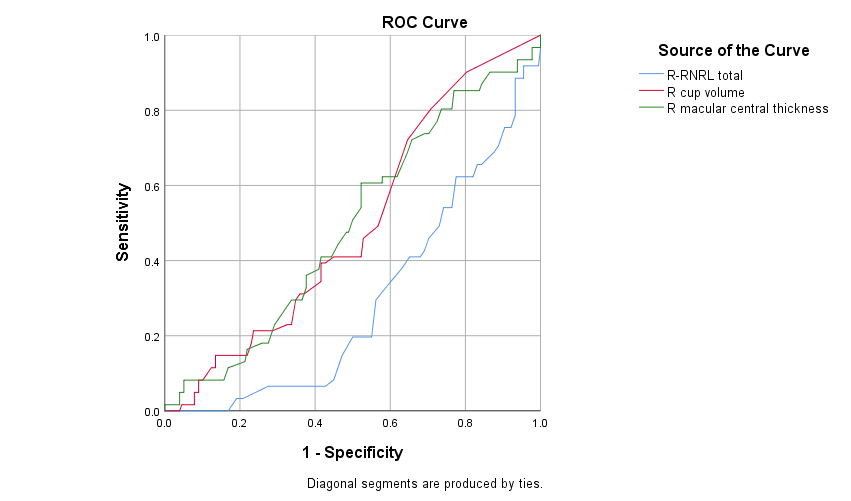


| **Area Under the Curve** | |
| --- | --- |
| Test Result Variable(s) | Area |
| R-RNRL total | .295 |
| R cup volume | .495 |
| R macular central thickness | .495 |
| The test result variable(s): R-RNRL total, R cup volume, R macular central thickness has at least one tie between the positive actual state group and the negative actual state group. Statistics may be biased. | |
