# Supplementary material for: Factors related to retinal nerve fiber layer thickness in bipolar disorder patients and major depression patients
Source: BMC Psychiatry. 2021 Jun 10;21:301. doi: 10.1186/s12888-021-03270-7 (PMC8191183; doi:10.1186/s12888-021-03270-7)
Supplement: Supplementary file 2 — Additional file 2. Major depression patient groups specificity, sensitivity, accuracy and ROC analysis. [file 12888_2021_3270_MOESM2_ESM.docx]

Depressed：


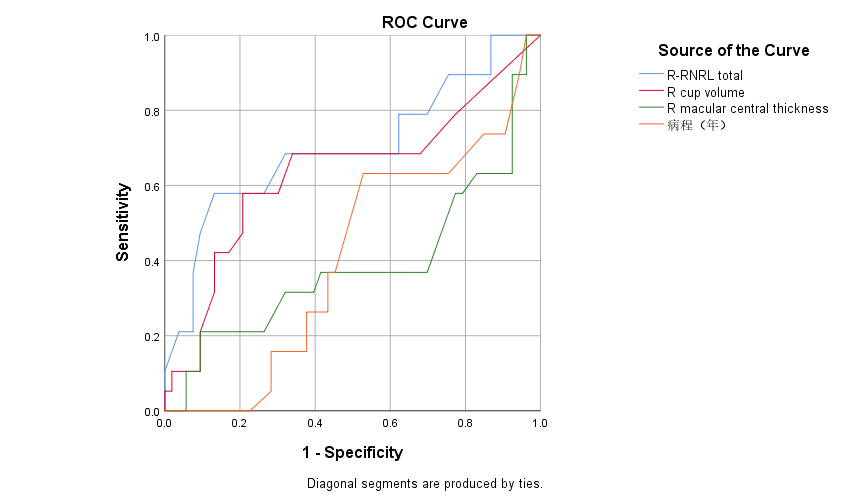


| **Area Under the Curve** | |
| --- | --- |
| Test Result Variable(s) | Area |
| R-RNRL total | .701 |
| R cup volume | .635 |
| R macular central thickness | .390 |
| Course of disease (years) | .412 |
| The test result variable(s): R-RNRL total, R cup volume, R macular central thickness, Course of disease (years) has at least one tie between the positive actual state group and the negative actual state group. Statistics may be biased. | |

Depressed:


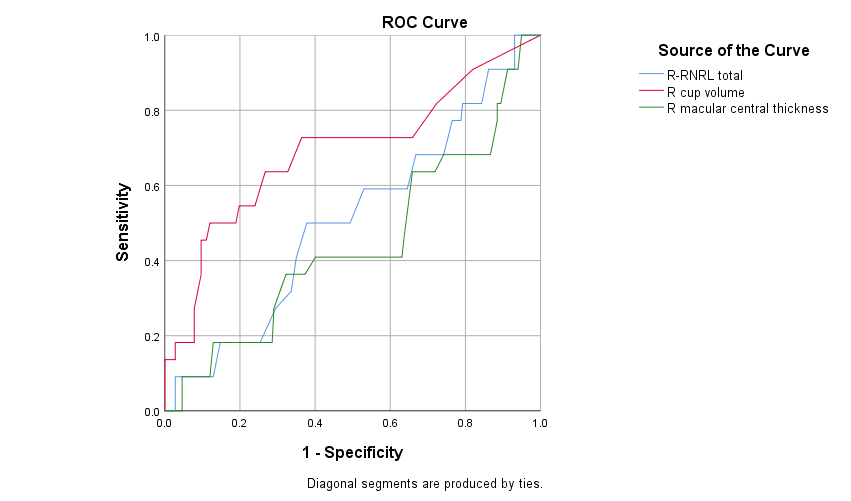


| **Area Under the Curve** | |
| --- | --- |
| Test Result Variable(s) | Area |
| R-RNRL total | .509 |
| R cup volume | .691 |
| R macular central thickness | .445 |
| The test result variable(s): R-RNRL total, R cup volume, R macular central thickness has at least one tie between the positive actual state group and the negative actual state group. Statistics may be biased. | |
